# Treatment of T1D via optimized expansion of antigen-specific Tregs induced by IL-2/anti-IL-2 monoclonal antibody complexes and peptide/MHC tetramers

**DOI:** 10.1038/s41598-018-26161-6

**Published:** 2018-05-25

**Authors:** Cristina Izquierdo, Angela Zarama Ortiz, Maximiliano Presa, Sara Malo, Anna Montoya, Nahir Garabatos, Conchi Mora, Joan Verdaguer, Thomas Stratmann

**Affiliations:** 1 Department of Cell Biology, Physiology and Immunology, Faculty of Biology, University of Barcelona, 08028 Barcelona, Spain; 2 Immunology Unit, Department of Experimental Medicine, School of Medicine, University of Lleida and IRB Lleida, 25008 Lleida, Spain; 3 CIBER of Diabetes and Associated Metabolic Diseases (CIBERDEM), Madrid, Spain; 4 Present Address: Otsuka Pharmaceutical, S.A, Barcelona, Spain; 5 Present Address: The Jackson Laboratory, Bar Harbor, USA; 6 Present Address: Danone Nutricia, Madrid, Spain; 7 Present Address: Benaroya Research Institute, Seattle, USA

## Abstract

Type 1 diabetes can be overcome by regulatory T cells (Treg) in NOD mice yet an efficient method to generate and maintain antigen-specific Treg is difficult to come by. Here, we devised a combination therapy of peptide/MHC tetramers and IL-2/anti-IL-2 monoclonal antibody complexes to generate antigen-specific Treg and maintain them over extended time periods. We first optimized treatment protocols conceived to obtain an improved islet-specific Treg/effector T cell ratio that led to the *in vivo* expansion and activation of these Treg as well as to an improved suppressor function. Optimized protocols were applied to treatment for testing diabetes prevention in NOD mice as well as in an accelerated T cell transfer model of T1D. The combined treatment led to robust protection against diabetes, and in the NOD model, to a close to complete prevention of insulitis. Treatment was accompanied with increased secretion of IL-10, detectable in total splenocytes and in Foxp3^−^ CD4 T cells. Our data suggest that a dual protection mechanism takes place by the collaboration of Foxp3^+^ and Foxp3^−^ regulatory cells. We conclude that antigen-specific Treg are an important target to improve current clinical interventions against this disease.

## Introduction

The role of regulatory T cells in type 1 diabetes (T1D) and their possible failure has been under debate. In the nonobese diabetic (NOD) mouse, a natural model with certain parallels to the human disease including the generation of autoreactive T and B cells specific for islet autoantigens^1^, the generation and the function of natural Foxp3 expressing regulatory T cells (Treg) have been studied. Beyond any doubt, these cells are crucial to prevent accelerated autoimmunity in this model^2,3^. Compared to T1D resistant strains, in NOD mice a reduction of this population was found by some groups^4,5^ but not corroborated by others^6^. This raised the question about the functionality of Foxp3^+^ Treg. Several reports showed that the suppressor activity of CD4^+^CD25^+^ T cells in the NOD strain was reduced^4^ and declined^7^ or met increasing resistance with age in the T effector population^8^. A comparative analysis between NOD and C57BL/6 (B6) mice showed that Foxp3^+^ Treg were equally functional in both strains^9^. However, the effector cells in the NOD strain were more difficult to control in comparison to the ones derived from B6 mice. We made similar observations by showing that oral tolerance induction in NOD mice failed with CTB-peptide fusion proteins while this was not the case in NODxB6 F1 mice^10^. A parallel observation about the difficulty to suppress effector T cells was made in human subjects where no difference in the frequencies of CD4^+^CD25^+^ between T1D patients and control subjects was detected^11^.

Nevertheless, it has been shown by several groups that the *in vitro* generation of Foxp3^+^ Treg and the subsequent adoptive transfer of these cells to NOD mice or the *in vivo* manipulation of Foxp3^+^ Treg can prevent T1D^12,13^. The most appropriate method to expand antigen-specific Treg *in vivo* remains an open debate, given the hypothesis that these are more potent to suppress organ-specific autoimmunity than nonspecific Treg^13,14^. The maintenance and expansion of the cells conferring acquired tolerance is a central issue. *In vivo* Ag-specific T cell expansion with regulatory function has been accomplished using MHC/peptide complexes^15^. For example, treatment with MHC/GAD peptide dimers prevented T1D via the generation of IL-10 producing antigen-specific Foxp3^−^ T cells without the de novo generation or expansion of Foxp3^+^ Treg^16^. On the other hand, Foxp3^+^ Treg can be expanded *in vivo* by treating mice with IL-2/anti-IL-2 mAb (IL-2:mAb) complexes^17^. The mAb JES6 recognizes an epitope of IL-2 that prevents it from binding to the low affinity IL-2 receptor composed of CD122 and γc, but allows IL-2 recognition by the high affinity receptor of IL-2, composed of CD122, γc and CD25, that is expressed constitutively by Foxp3^+^ Treg^18^. The expansion of polyclonal Treg by means of these complexes successfully prevented autoimmunity in an EAE model and also supported islet allograft survival^17^.

We therefore wondered whether a combined treatment with MHC/peptide molecules and IL-2:mAb complexes might lead to the expansion of Foxp3^+^ antigen-specific regulatory T cells, and asked to what extent this treatment might serve to prevent disease in NOD mice. Here, we employed a mimotope peptide, 2.5 mi^19^, complexed to A^g7^, the MHC class II allele expressed by NOD mice. A^g7^/2.5 mi tetramers detect a natural CD4 T cell population that shares Ag-recognition with the diabetogenic T cell clone BDC-2.5. This T cell population, termed 2.5 mi^+^ T cells, is generated early during life and depends on the selection by A^g7 ^^19^. The natural antigen recognized by BDC-2.5, and by analogy by 2.5 mi^+^ T cells, has recently been identified as chromogranin A^20^. It was subsequently shown that the epitope, WE14, is best recognized after enzymatic modification^21^, and that this modified peptide is also recognized by CD4 T cells derived from T1D patients^22^. A further study has now shown that BDC-2.5 recognizes a hybrid peptide containing amino acid sequences from insulin as well as from chromogranin A^23^.

Here, we show that the combined treatment with A^g7^/2.5 mi tetramers and IL-2:mAb complexes leads to a potent prevention of T1D. The treatment leads to a large expansion of Ag-specific Foxp3^+^ Treg that acquire markers of activation, suppression and homing, and is accompanied by the proliferation of an antigen-specific Foxp3^−^ population that produces anti-inflammatory IL-10.

## Materials and Methods

### Reagents and cells

Antibodies used for flow cytometry were purchased from BioLegend (San Diego, CA, USA). Streptavidin-PE and allophycocyanin were obtained from Columbia Biosciences (Columbia, MD, USA). IL-2 cytokine was expressed in and purified from *E. coli*. To ensure low endotoxin levels (<0.1 EU/µg of protein) ToxinEraser Endotoxin Removal kit (GenScript, Piscataway, NJ, USA) was used. JES6–1A12 anti-IL-2 monoclonal antibody was purchased from Bio X Cell (West Lebanon, NH, USA). 2.5 mi synthetic peptide (AHHPIWARMDA) was purchased from GL Biochem (Shangai, China). Unless otherwise mentioned, all other reagents were obtained from Fluka or Sigma-Aldrich (Madrid, Spain).

### Mouse strains

NOD/LtJ, B6.Foxp3^EGFP^ ^24^, NOD.SCID and BDC-2.5/N TCR Tg mice were originally purchased from The Jackson Laboratory (Bar Habor, ME, USA) and further bred in our special pathogen-free animal facility (Parc Científic Barcelona, Spain). NOD.Foxp3^EGFP^ mice were generated in-house using a speed congenic approach by backcrossing B6.Foxp3^EGFP^ mice into the NOD background while monitoring 15 independent *Idd* loci. Mice homozygous after four generations for every single *Idd* locus were further backcrossed for six more generations with NOD mice and then intercrossed to obtain homozygous NOD.Foxp3^EGFP^ (F10) mice. Unless specifically mentioned in the text, mice were between 8 and 14 weeks of age for experimental use. Animal studies were approved by the corresponding animal care committees of the Autonomic Government of Catalonia and are in accordance with the current national regulation.

### Generation of recombinant proteins

MHC-peptide tetramer complexes were prepared as previously published^19^. In brief, *Drosophila melanogaster*-derived SC2 cells were transfected with DNA plasmids coding for the A^d^ MHC α-chain containing a biotinylation sequence and the A^g7^ MHC β-chain tethered N-terminally to the 2.5 mimotope or the glucose-6-phosphate isomerase (GPI) 282–292 control peptide^19^ along with a plasmid conferring puromycin resistance. Molecules purified from culture supernatants were biotinylated using the BirA enzyme as published. Fluorescent tetramers were generated by incubation of A^g7^ monomers with PE or allophycocyanin-labeled streptavidin in 5:1 molar ratio.

For protein immunization, A^g7^/2.5 mi tetramers were prepared by incubating 25 µg/dose of A^g7^/2.5 mi monomers with sterile avidin at 5:1 molar ratio during 1 h at 37 °C. IL-2:mAb complexes were prepared by incubating 1 µg of IL-2 with 5 µg of JES6–1A12 anti-IL-2 antibody (Bio X Cell, West Lebanon, NH, USA) during 1 h at RT.

### Flow cytometry analysis

Single cell suspensions of spleen and lymph nodes were generated by mechanical disruption of the corresponding organs in PBS-2% FBS. Pancreatic islets were isolated by the intraductal collagenase technique^25^ and purified by several hand-picking stages in HBSS medium. Islets were then disrupted with a tissue pestle and left more than an hour at 37 °C in RPMI complete medium to allow cell exit. Single cell suspension of lymphocytes was later obtained after decanting large undigested tissue.

Antigen-specific T cell analysis was carried out as previously described^10^. Briefly, single cell suspensions were blocked with avidin (Sigma-Aldrich, Madrid, Spain) in PBS-2% FBS and stained at 4 °C during 1 h with PE- and/or APC-labeled A^g7^/2.5 mi tetramers or A^g7^/GPI tetramers as negative control. Depending on the combination of surface marker analysis, Pacific Blue- or Allophycocianin-anti-CD4, PECy7-anti-ICOS, APC-anti-CD25, PECy7-anti-GITR, APC-anti-CXCR3 and PECy5 anti-CD8, anti-CD11c, anti-CD19 and anti-F4/80 (dump channel) were used (BioLegend, CA, USA). Foxp3 was detected via enhanced green fluorescence protein (EGFP) expression. Dead cells were excluded by addition of 5 µg/ml of propidium iodide immediately before acquisition (see supplemental Fig. 1 for gating strategy). Flow cytometry was performed using FACScanto II, LSR II, FACSAria II instruments (Becton Dickinson Immunocytometry Systems, Mountain View, CA, USA). Data were analyzed using FlowJo software (Tree Star Inc, Ashland, OR, USA).

### Combined tetramer and IL-2:mAb complex treatment and insulitis score analysis

For diabetes prevention studies, 5 to 6-weeks-old NOD.Foxp3^EGFP^ females were treated until week 35 of age and maintained until week 45 of age. Each mouse received i.p. doses of 100 µl of the corresponding protein: for A^g7^/2.5 mi tetramer, 25 µg of total peptide:MHC monomer per dose; for IL-2:mAb complexes, 1 µg of IL-2 complexed with 5 µg of anti-IL-2 mAb (clone JES6–1A12) per injection. For the tetramer + IL-2:mAb complexes treatments (Combined Intensive and Simple treatments), females received during the first 8 days the combined optimized treatment (see schematic diagram on Fig. 3C) and on the following weeks a maintenance dose of IL-2:mAb either once (for the combined simple treatment) or twice a week (for the combined intensive treatment), and once every two weeks one dose of A^g7^/2.5 mi tetramer. Animals not receiving the combined treatments (i.e. that received only tetramer or IL-2:mAb complexes) were immunized following the same regime as the intensive combined treatment but the respective reagents were omitted (see Supplemental Fig. 7 for a schematic diagram).

Levels of glycaemia were determined weekly starting at week 10 of age using an ACCU-CHEK Aviva system (Roche Diagnostics, Switzerland). Animals were considered diabetic after two consecutive blood glucose readings above 200 mg/dL.

Insulitis scoring was performed on hematoxylin-stained pancreatic sections of surviving diabetes-free females at the end-point of the incidence experiment. At least 100 islets were pooled from different sections of pancreata obtained from three different mice per group.

### *In vitro* Treg suppression assays

T effector (Teff) cells (CD11c^−^ CD19^−^ CD8^−^ F4/80^−^ CD4^+^ Foxp3^EGFP−^) were sorted from the spleen of naive NOD.Foxp3^EGFP^ mice (8 to 11 weeks-old) and stained with CellTrace Violet Cell Proliferation Kit (Molecular Probes, Life technologies, USA). In brief, cells were washed once with cold PBS, resuspended in 1 ml of 1 μM CellTrace Violet solution/10^6^ cells and incubated for 20 minutes at 37 °C in the dark. Staining was quenched by adding five times the original staining volume of culture medium (containing 10% of FBS) to the cells and by incubating for 5 minutes at RT. Cells were pelleted by centrifugation, resuspended in fresh pre-warmed complete RPMI medium and used in the suppression assay.

Sorted splenic CD11c^−^ CD19^−^ CD8^−^ F4/80^−^ CD4^+^ Foxp3^EGFP+^ cells (Treg) from 8 to 11 weeks-old NOD.Foxp3^EGFP^ mice treated as referred were incubated at different Teff:Treg ratios (1:1; 1:0.5; 1:0.25, 1:0.125 or 1:0) with 5 × 10^4^ CD4^+^ Foxp3^EGFP−^ cells (Teff) stained with CellTrace Violet dye. Mitomycin C treated splenocytes isolated from 10 weeks-old naive NOD.SCID mice were used as accessory cells at 2 × 10^5^ cells per well. Cells were maintained in 96-well round-bottom plates for 72 h at 37 °C in 5% CO_2_ in the presence of soluble α-CD3 (1 µg/ml) in RPMI complete medium containing 10% of fetal bovine serum, 10 mM HEPES, 2 mM L-Glutamine, 1 × Antibiotic/antimicotic solution (Biowest, Nuaillé, France) and 50 µM 2-mercaptoethanol.

The calculation of cell division percentage of Teff within the Teff:Treg co-cultures was performed using FlowJo software (Tree Star Inc, Ashland OR, USA). The proliferation obtained in 1:0 wells was considered 100% of division of Teff.

### Adoptive transfer experiments

For the T1D accelerated model, 1 × 10^6^ naive splenocytes from 11 to 14 weeks-old BDC-2.5/N TCR Tg mice were co-transferred i.p. into young NOD.SCID mice (4–5 weeks-old) with 1 × 10^7^ splenocytes isolated from 8 to 13 weeks-old NOD.Foxp3^EGFP^ females treated as mentioned. Diabetes onset was assessed starting at 7 days post-transfer. Healthy animals were maintained up to 119 days after-transfer.

### Cytokine quantification

Total splenocytes (5 × 10^5^ cell per well) isolated from NOD.Foxp3^EGFP^ females treated as mentioned in the text were stimulated for 72 h in complete RPMI + 10% FBS in the presence of the correspondent stimuli (2.5 mi synthetic peptide [10 µM], Concavalin A [2.5 µg/ml] or anti-CD3 antibody [2 µg/ml]). Culture supernatants were collected after the stimulation period and IL-10 and IFN-γ secretion were detected by ELISA following the manufacturer’s instructions (mouse IL-10 ELISA Ready-SET-Go [2^nd^ Generation] and mouse IFN-γ ‘Femto-HS’ High Sensitivity ELISA Ready-Set-Go; eBioscience).

### Statistical analysis

The data are presented as mean ± SEM. Statistical analysis was performed using Graph Pad Prism software 5.0c (San Diego, CA, USA). One-way ANOVA or *t* test were applied wherever appropriate. A *p* value < 0.05 was considered significant.

Cumulative incidence of diabetes was determined by Kaplan-Meier estimates using GraphPad Prism software and curves were analyzed by log-rank test for statistical differences.

### Data availability

The datasets generated or analysed during this study are available from the corresponding author on reasonable request.

## Results

### Expansion of 2.5 mi^+^ T cells with IL-2:mAb complexes

**Figure 1:**
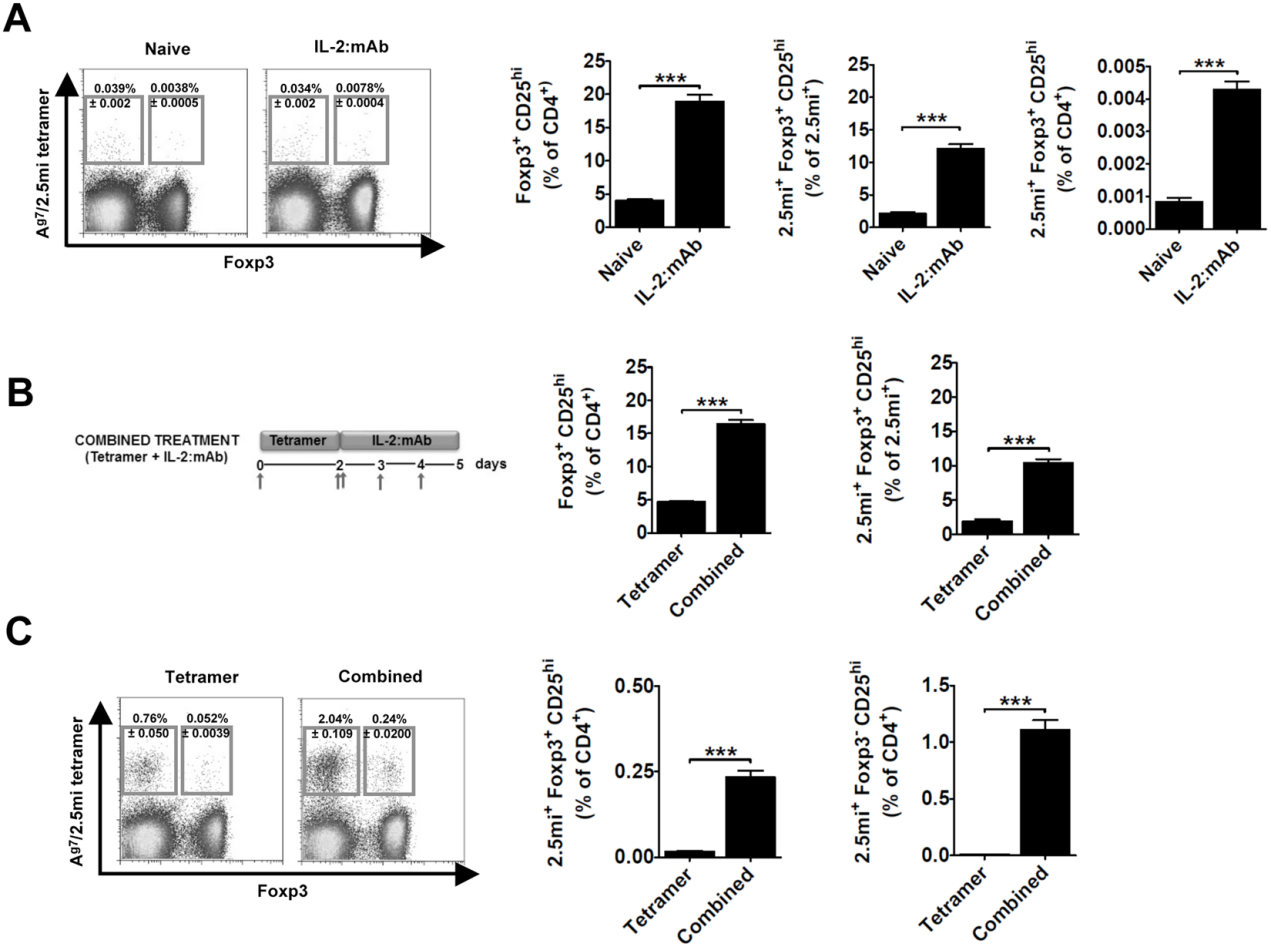
The administration of a combined treatment of A^g7^/2.5 mi tetramers and IL-2:mAb complexes increases the percentage of antigen-specific regulatory T cells in NOD.Foxp3^EGFP^ mice. Analysis of the spleen are shown. Ten to twelve weeks-old NOD.Foxp3^EGFP^ mice were injected i.p. with 3 daily doses (days 0, 1, 2) of IL-2:anti-IL-2 mAb JES6–1A12 (IL-2:mAb; complex ratio 1:5 µg) or left untreated (n = 9 per experimental group). Spleens were removed at day 3 and analyzed by flow cytometry. (**A**, left) Representative flow cytometric profiles showing 2.5 mi^+^Foxp3^+^ (Treg) and 2.5 mi^+^Foxp3^−^ (Tconv) T cell percentages ± SEM within total CD4^+^ T cells. (**A**), center panels) Expansion of polyclonal and 2.5 mi^+^ CD25^high^Foxp3^+^CD4^+^ T cell populations within total CD4^+^ (left) or antigen-specific CD4^+^ T cells (right). (**A**, right) Bar graph indicating the percentage of 2.5 mi^+^Foxp3^+^CD25^high^ T cells found in spleen after each treatment. (**B**) Schematic diagram of the combined treatment based on the administration of A^g7^/2.5 mi tetramers and IL-2:mAb complexes (left). Arrows indicate days of protein injection. 25 µg of tetramer and 1:5 µg of IL-2:mAb complexes are injected i.p. per mouse and dose. Ten to 12 weeks-old NOD.Foxp3^EGFP^ mice were treated as shown or with A^g7^/2.5 mi tetramer only (n = 9–12 animals per group). Spleens were harvested on day 5 and cells analyzed by flow cytometry. Analysis of total Foxp3^+^CD25^hi^ cells within total CD4 T cells (B, middle) and of 2.5 mi^+^Foxp3^+^CD25^hi^ CD4 T cells within 2.5 mi^+^ T cells (B, right) is shown. (**C**) Left panel, representative dot plot data showing splenic 2.5 mi^+^Foxp3^+^ (Treg) and 2.5 mi^+^Foxp3^−^ (Tconv) T cell percentages ± SEM after combined or tetramer only treatment. Middle and right panel, analysis of 2.5 mi^+^ CD25^hi^CD4^+^Foxp3^+^ and 2.5 mi^+^ CD25^hi^CD4^+^Foxp3^−^ T cells within total CD4^+^ T cells is shown. For the analysis, cells were gated on CD19^−^, CD8^−^, CD11c^−^, F4/80^−^, propidium iodide (PI)^−^, CD4^+^ and CD25^hi^. Errors bars indicate mean ± SEM. The *p* values were determined by Student t test. ***p < 0.0005. Graphs represent a compilation of 3–4 independent experiments. No A^g7^/GPI-specific T cell expansion was observed in naive or treated mice analyzed in this figure.

**Figure 2:**
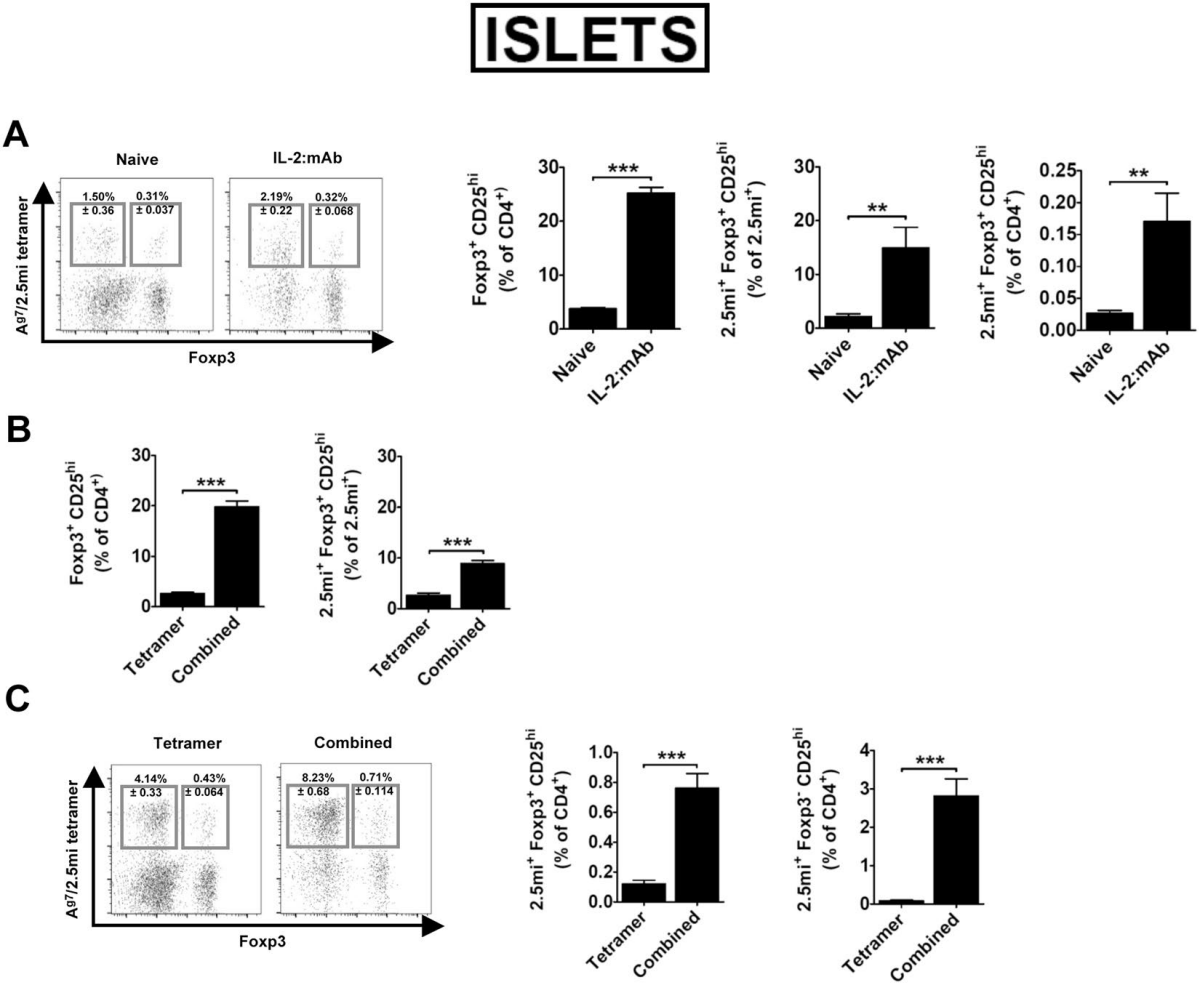
The administration of the combined treatment increases the percentage of antigen-specific CD25^hi^ regulatory T cells in islets of NOD.Foxp3^EGFP^ mice. The same animals as in Fig. 1 were analyzed to determine the percentage of CD25^hi^ T cells in pancreatic islets of treated mice. Please refer to Figure legend 1 for a detailed panel description.

A promising approach to treat autoimmunity is the generation of Ag-specific regulatory T cells. Previous reports have described the selective expansion of Foxp3^+^CD4 T cells via treatment with IL-2 complexed to the anti-IL-2 mAb JES6-1 (IL-2:mAb)^12,17,26,27^. We were curious to see whether and to which extent these complexes could affect Foxp3^+^2.5 mi^+^ T cells, given their presence in NOD mice^10^. Foxp3^+^2.5 mi^+^ T cells within the CD4 T cell population in NOD.Foxp3^EGFP^ mice, injected 3 consecutive days with IL-2:mAb, approximately doubled in the spleen (Fig. 1A, left). In these mice, Foxp3^+^CD25^hi^CD4 T cells more than quadruplicated compared to naive animals. A similar observation was made within the 2.5 mi^+^ T cell population where cell numbers of Foxp3^+^CD25^hi^ T cells increased more than fourfold in spleen (Fig. 1A, center panels) and islets (Fig. 2A, center panels). A fourfold and sixfold increase of Foxp3^+^CD25^hi^2.5 mi^+^ T cells was also established in spleen and islets, respectively (Fig. 1A, right and Fig. 2A, right; see Supplementary Fig. 1 for gating strategy), in comparison to total CD4 T cells, however, given the low frequency of these T cells in naive mice, the increase was minimal especially in the spleen, albeit statistically significant.

Previous experiments in our lab have shown that treatment of NOD mice with A^g7^/2.5 mi tetramers leads to the expansion of 2.5 mi^+^ T cells (unpublished), similar to what has been shown by Tisch and colleagues for GAD65-derived peptides complexed to this MHC class II allele^16^. We thus decided to test whether treatment with A^g7^/2.5 mi tetramers could be combined with IL-2:mAb complexes to increase the Foxp3^+^2.5 mi^+^ T cell population *in vivo*. NOD.Foxp3^EGFP^ mice were injected with A^g7^/2.5 mi tetramers at day 0 and day 2, and IL-2:mAb complexes at days 2–4, an immunization protocol termed “combined treatment” from here onwards (Fig. 1B, left). T cells were analyzed at day 5 at which point maximal Foxp3^+^ T cell expansion has been reported^17^. Polyclonal Foxp3^+^CD25^hi^ Treg rose from 5% to over 15% in mice treated with IL-2:mAb complexes and tetramers, compared to tetramers only, in spleen and from 3% to 20% in islets. Within the 2.5 mi^+^ T cell population, percentages of Foxp3^+^CD25^hi^ Treg rose three- to fivefold in islets and spleen, respectively (Fig. 1B, right, and Fig. 2B). Tetramer treatment combined with IL-2:mAb complexes led to a strong expansion of both Foxp3^+^ and Foxp3^−^ 2.5 mi^+^ T cells (Fig. 1C, left), and compared to naive mice (Fig. 1A, left) the expansion of Foxp3^+^2.5 mi^+^ T cells increased approximately 70-fold in the spleen and over two fold in the islet (Fig. 2C, left). Significantly more 2.5 mi^+^Foxp3^+^CD25^hi^ T cells were generated in both tissues by the combined treatment compared to tetramer-treatment only (Figs 1C and 2C, middle, and Supplementary Tables 1 and 2). However, the undesired expansion of activated 2.5 mi^+^ Foxp3^−^CD25^hi^ T cells in spleen (Fig. 1A, right) and islets (Fig. 2A, right), presumably composed at least in part of effector T cells, prompted us to investigate further.

**Figure 3:**
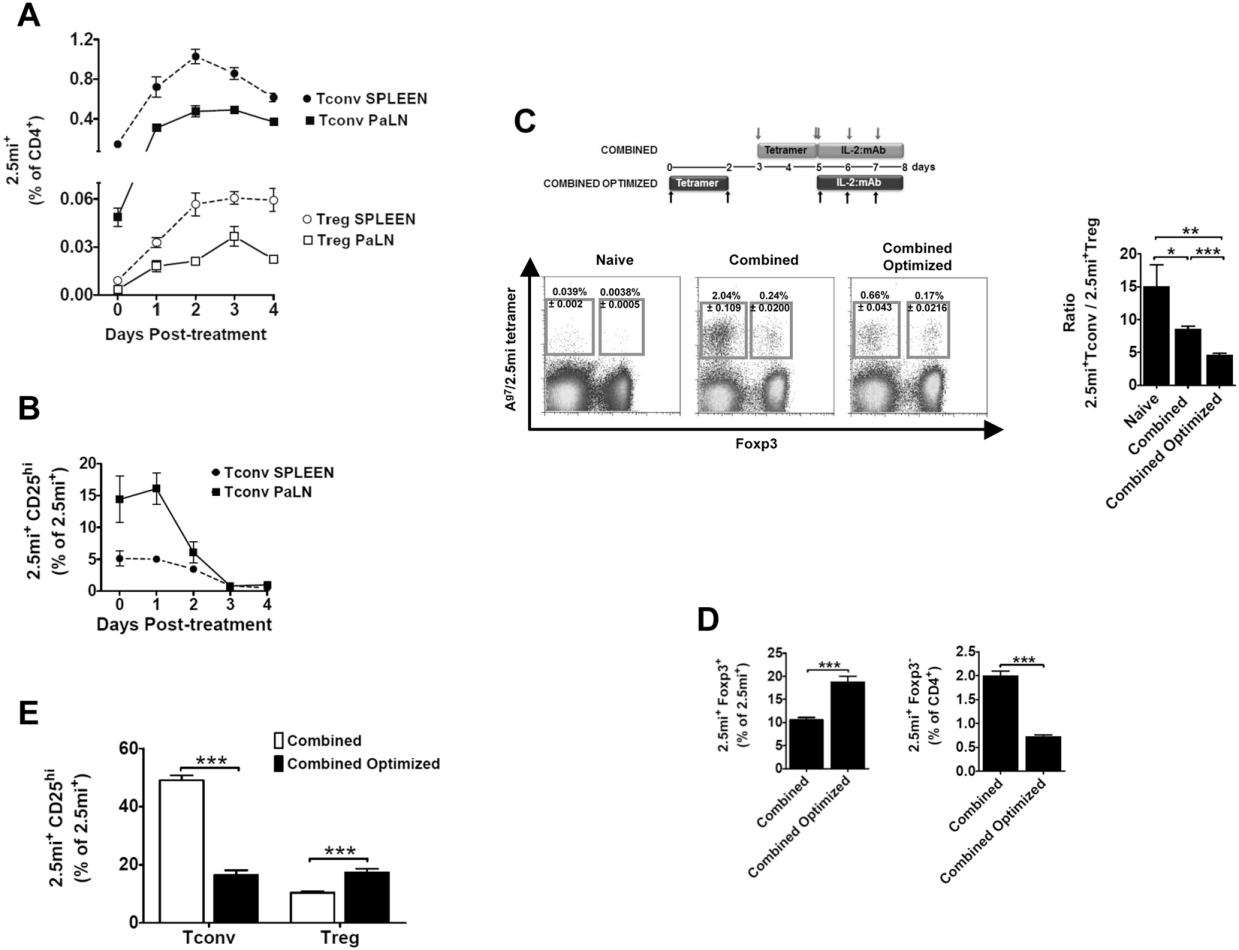
Combined optimized treatment of A^g7^/2.5 mi tetramer and IL-2:mAb complexes improves the expansion of 2.5 mi^+^ Treg cells and controls the increase of antigen-specific Foxp3^−^ T cells in NOD.Foxp3^EGFP^ mice. Unless otherwise indicated in the figure, analysis of spleen are shown. Seven to 14 weeks-old NOD.Foxp3^EGFP^ mice were injected i.p. with two doses of A^g7^/2.5 mi tetramer (25 µg/dose) at day −2 and day 0 and sacrificed on day 0, 1, 2, 3 and 4 after the last injection. Spleen and pancreatic lymph nodes (PaLN) were harvested and the 2.5 mi^+^ T cell response was analyzed by tetramer staining. (**A**) Expansion and contraction of antigen-specific Tconv (T CD4^+^2.5 mi^+^Foxp3^−^) and Treg (T CD4^+^2.5 mi^+^Foxp3^+^) populations in the extracted organs. (**B**) Time-course of the CD25 expression on CD4^+^2.5 mi^+^Foxp3^−^ (Tconv) cells. Mean ± SEM of 4 independent experiments (2–3 animals per time point) is shown in graphs A and B. (**C**) Top, schematic representation of the combined and the combined optimized treatment with A^g7^/2.5 mi tetramers and IL-2:mAb complexes. Ten to 12 weeks-old NOD.Foxp3^EGFP^ mice were treated as indicated in the schema. T cell responses were determined at day 8 of treatment. Bottom left, representative dot plots showing the 2.5 mi^+^ Tconv and Treg cell percentages ± SEM within total CD4^+^ T cells in the spleen. Bottom right, ratio of Tconv/Treg in Ag-specific T cells in spleen of mice treated as indicated. (**D**) Percentages of 2.5 mi^+^Foxp3^+^ cells within the total Ag-specific population (left) and of 2.5 mi^+^Foxp3^−^CD4^+^ T cells within CD4 T cells (right) found in spleen after immunizing mice with the combined and the combined optimized treatments. (**E**) Percentages of activated (CD25^hi^) antigen-specific Tconv (Foxp3^−^) and Treg (Foxp3^+^) cells within the 2.5 mi^+^ T cell population in the spleen. (**C,D)**, and (**E)**: Error bars in graphs indicate mean ± SEM of 3 to 4 independent experiments including 2–3 animals per group of each experiment. The *p* values result from a Student t test analysis. **p < 0.005, ***p < 0.0005. No A^g7^/GPI-specific T cell expansion was observed in naive or treated mice analyzed in this figure.

**Figure 4:**
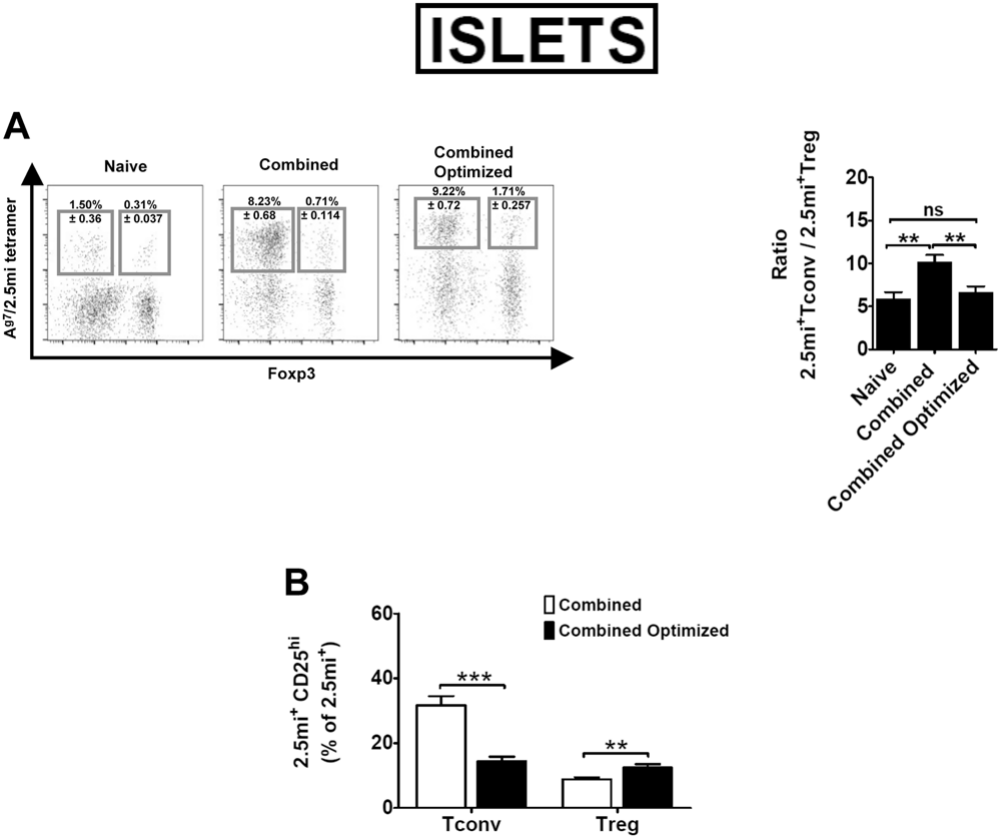
Combined Optimized treatment improves the expansion of 2.5 mi^+^ Treg cells and controls the increase of antigen-specific Foxp3^−^ T cells in NOD.Foxp3^EGFP^ mice in pancreatic islets. The same animals used in Fig. 3 C–E were used for the analysis of 2.5 mi^+^ T cells in pancreatic islets. (**A**) Left, representative dot plots depicting Ag-specific Tconv and Treg percentages ± SEM within total CD4^+^ T cells in islets. Right, bar diagram showing the ratio of Tconv/Treg in 2.5 mi^+^ T cells. (**B**) Percentages of activated (CD25^hi^) antigen-specific Tconv (Foxp3^−^) and Treg (Foxp3^+^) cells within the 2.5 mi^+^ T cell population in islets. Error bars in graphs indicate mean ± SEM of 3 to 4 independent experiments including 2-3 animals per group. The *p* values result from a Student t test analysis. **p < 0.005, ***p < 0.0005. No A^g7^/GPI-specific T cell expansion was observed.

JES6-1 blocks adhesion of IL-2 to the low affinity receptor composed of the β and γc chain (IL-2Rβγ)^18,28^. Therefore, IL-2:mAb complexes generated with this Ab target the α-chain (CD25) of the high affinity IL-2 receptor, expressed constitutively by Foxp3^+^ Treg^29^, but also by activated CD4 conventional T cells (Tconv) that consume IL-2 during their expansion phase^30^. In order to limit IL-2 availability to Foxp3^−^ Treg cells within the 2.5 mi^+^ T cell population, we analyzed the expansion of 2.5 mi^+^Foxp3^+^ and 2.5 mi^+^Foxp3^−^ T cells as well as their CD25 expression in NOD.Foxp3^EGFP^ mice that had been treated with A^g7^/2.5 mi tetramers at day −2 and day 0. Maximal expansion of Foxp3^−^2.5 mi^+^ T cells was observed in spleen and pancreatic LN at day 2, while Foxp3^+^2.5 mi^+^ T cells peaked in these LN at day 3 (Fig. 3A). CD25 expression by conventional 2.5 mi^+^ T cells peaked at day 1 in pancreatic LN and rapidly declined to reach minimal values starting at day 3. In the spleen, highest values were observed at day 0, after which they steadily declined (Fig. 3B). We thus predicted that an optimal effect of IL-2:mAb complexes treatment should be obtained by injection on day 3, when maximal Treg expansion is observed in spleen and pancreatic LN, while CD25 expression has diminished to minimal levels in the Tconv population. We compared therefore the effect of IL-2:mAb complexes when given during the expansion phase of 2.5 mi^+^ T cells (combined treatment) in comparison to day 3 after the last A^g7^/2.5 mi tetramer dose (combined optimized treatment, Fig. 3C). Both groups were analyzed the following day of the last IL-2:mAb complex dose. Indeed, in the spleen the combined optimized treatment resulted in close to 20% of Foxp3^+^ T cell within 2.5 mi^+^ T cells, compared to 10% after the combined treatment, a significant difference. The combined optimized treatment further led to a reduced expansion of 2.5 mi^+^ Tconv and therefore a decrease of the Tconv/Treg ratio (Fig. 3C,D and see supplementary Fig. 2 for absolute cell numbers). In spleen, significantly more 2.5 mi^+^ Tconv cells expressed CD25, i.e. were more activated, following the combined treatment compared to the combined optimized treatment, while the opposite occurred in the Foxp3^+^2.5 mi^+^ T cell population (Fig. 3E and Supplementary Tables 1 and 2). An overall similar observation was made in the islets (Fig. 4). Taken together, the combined optimized treatment favored the expansion of antigen-specific Treg as well as their activation.

### Phenotypical analysis of expanded regulatory T cells/Enhanced suppressor activity

**Figure 5:**
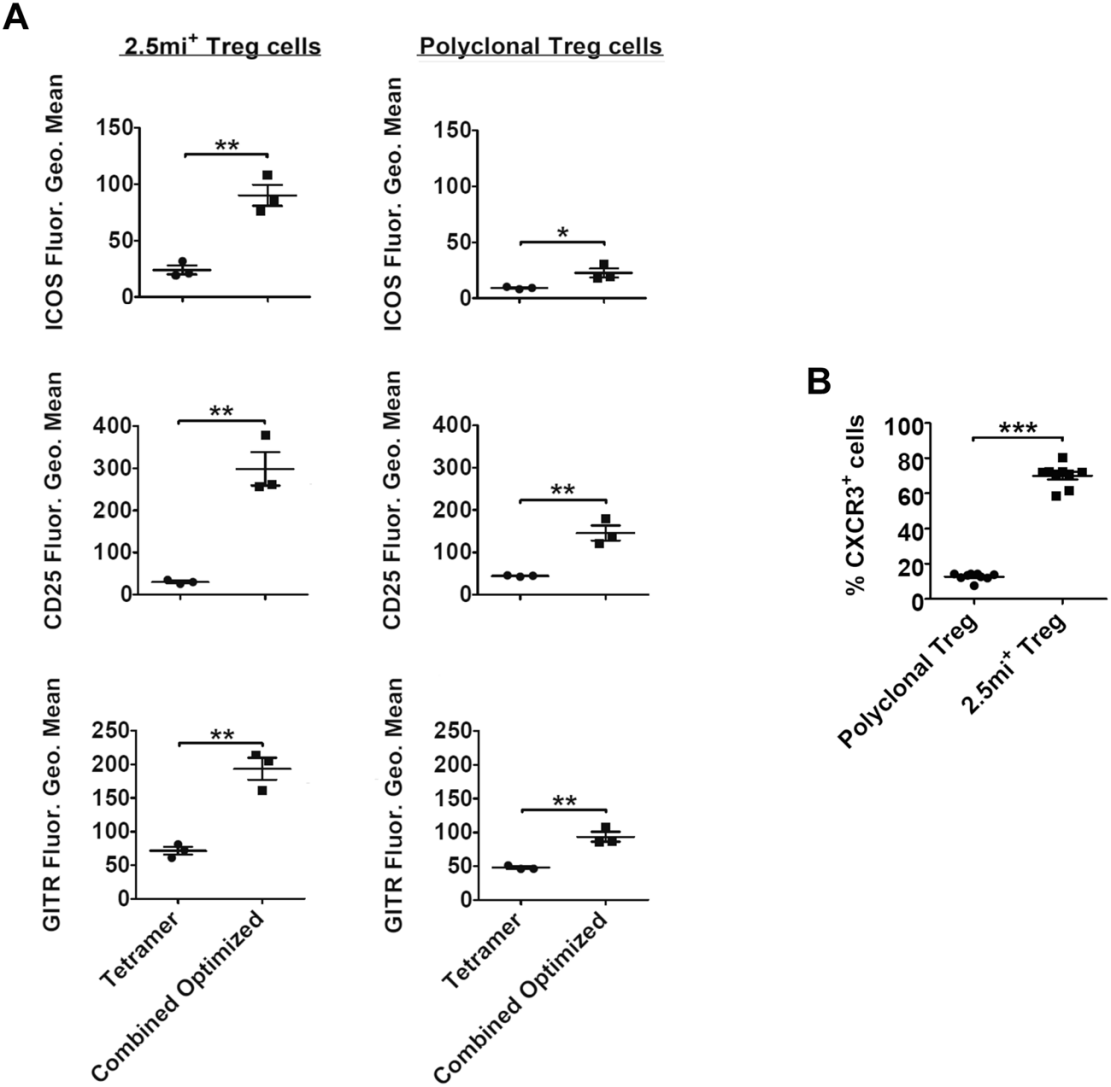
Phenotype surface markers on regulatory T cells expanded with the combined optimized treatment. Analysis of the spleen is shown. NOD.Foxp3^EGFP^ females received either the combined optimized treatment or A^g7^/2.5 mi tetramer injections only. Spleens were isolated at day 8 of treatment and used for the determination of: (**A**) the MFI of ICOS, CD25 and GITR, on the surface of 2.5 mi^+^ (left) and polyclonal (right) Foxp3^+^CD4^+^ T cells from treated animals; (**B**) the percentages of CXCR3^+^ cells within antigen-specific (right) or polyclonal (left) Foxp3^+^CD4^+^ T populations. 2.5 mi^+^ cells were excluded from polyclonal T cells for all the analysis. Data shown in (**A**) is a representative example from 3 independent experiments; n = 2-3 animals/experimental group. Data in (**B**) is a compilation of 3 independent experiments with 2-3 animals per treatment group. No A^g7^/GPI-specific T cell expansion was observed in any experiment.

The enhanced expression of CD25, ICOS and GITR indicate an improved suppressor activity by Foxp3^+^ Treg^17,31,32^. We investigated inasmuch the combined optimized treatment affected total and Foxp3^+^2.5 mi^+^ Treg in comparison to treatment with A^g7^/2.5 mi tetramers only. In the spleen, all three surface markers were upregulated following the combined optimized treatment compared to tetramer treatment only, especially in 2.5 mi^+^ but also in total Foxp3^+^ Treg (Fig. 5A and Supplementary Fig. 3A). CXCR3 is expressed by Th1 cells that participate in T1D pathogenesis, and is implicated in the homing of these cells to the inflamed tissues. Foxp3^+^ Treg expressing the same chemokine receptor therefore are expected to home to the same tissues, allowing them to suppress at these locations pathogenic T cells^32^. Further, CXCR3^+^ Treg reportedly actively participate in islet protection^33^. Expanded 2.5 mi^+^ Treg were in their vast majority CXCR3 positive in the spleen, while only a small proportion of polyclonal Foxp3^+^ Treg expressed this receptor (Fig. 5B and Supplementary Fig. 3B). Compared to naive NOD.Foxp3^EGFP^ mice, percentages of polyclonal Foxp3^+^ Treg in the islets augmented less than 2-fold but more than 7-fold in the case of Foxp3^+^2.5 mi^+^ T cells following the combined optimized treatment (Supplementary Table 3). Therefore, the combined optimized treatment led to an increment of antigen-specific Treg within the polyclonal Treg population.

**Figure 6:**
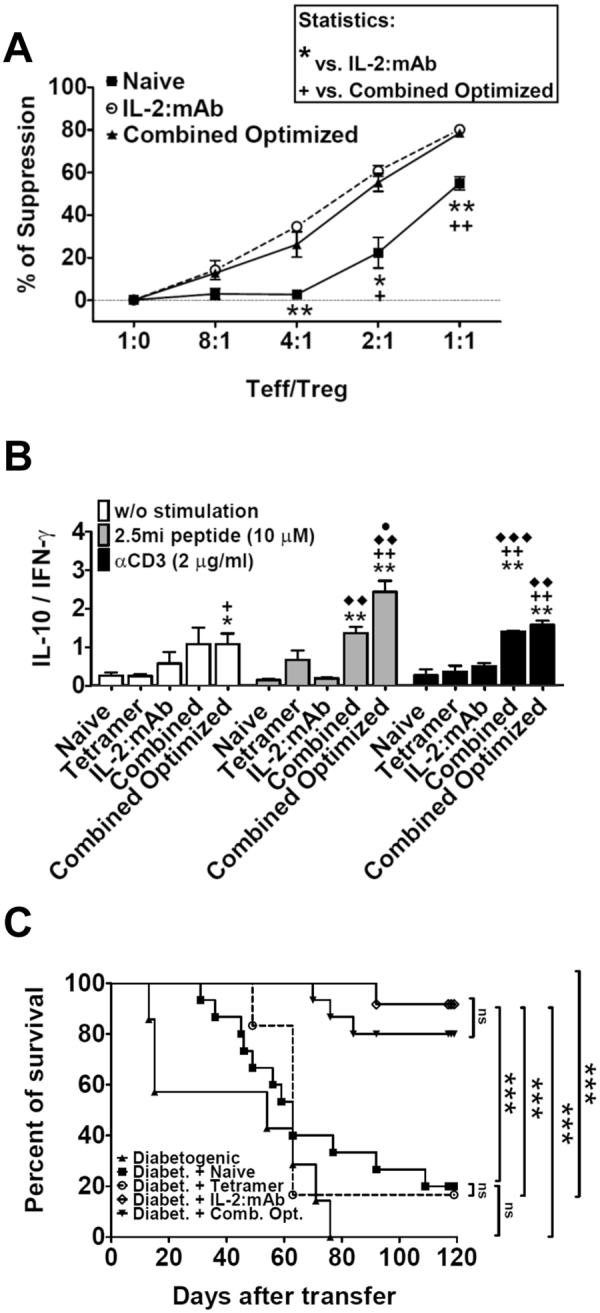
Regulatory T cells expanded with the combined optimized treatment show superior suppressive function, both *in vitro* and *in vivo*; and exhibit enhanced secretion of the anti-inflammatory cytokine IL-10. Analysis of spleen is shown. As in Fig. 3, NOD.Foxp3^EGFP^ females received either the combined optimized treatment, the combined treatment, A^g7^/2.5 mi tetramer only, IL-2:mAb complexes only or were left untreated. Spleens were isolated at day 8 of treatment and used for the subsequent experiments. (**A**) *In vitro* suppression of CD4^+^Foxp3^−^ T cell proliferation by sorted CD4^+^Foxp3^+^ Treg cells isolated from mice treated as mentioned above. Proliferation was measured by flow cytometry via CellTrace Violet dye dilution after 3 days of co-culture at the indicated population ratio in the presence of anti-CD3 antibody as stimulus. Student t test (95% confidence) was applied for the calculation of statistical differences between naive and treated groups. The results of one of two independent experiments are shown (n = 3 per point). (**B**) Isolated splenocytes from treated (as referred) NOD.Foxp3^EGFP^ females were seeded into 96-well culture plates without stimulation or with the addition of 2.5 mi peptide or anti-CD3 antibody. Culture supernatants were collected at day 3 and assessed by ELISA for the presence of IL-10 and IFN-γ. The ratio between IL-10 secretion and IFN-γ production is shown for each condition. Data represent a compilation of three independent experiments. Statistical analysis (Student t test, 95% confidence): *, vs. naive; +, vs. only tetramer treatment; ♦, vs. IL-2:mAb complexes treatment; ●, vs. combined treatment. (**C**) Total splenocytes harvested from treated NOD.Foxp3^EGFP^ females were transferred along with splenocytes from naive BDC-2.5 transgenic females to young NOD.SCID recipients (spleens from treated NOD.Foxp3^EGFP^ female mice were extracted on day 8 after treatment as shown in Fig. 3C for individuals receiving tetramer only and the combined optimized treatment or on the day following the last dose of IL-2:mAb complexes for individuals receiving IL-2:mAb complexes only). Diabetes onset was determined by blood glucose measurements (animals with two consecutive measures above 200 mg/dl were considered diabetic). Percent of diabetes-free mice is depicted. Mantel-Cox test was applied to evaluate statistical differences between groups (n = 6-15/group).

Sorted total Foxp3^+^ T cells isolated from mice receiving either the combined optimized treatment or the IL-2:mAb complex treatment only had an enhanced suppressor activity in comparison to Foxp3^+^ T cells isolated from naive animals (Fig. 6A). The anti-inflammatory cytokine IL-10 is secreted by various cells types including Tr1 cells and Foxp3^+^ Treg. Maximal IL-10 secretion was observed by total splenocytes from mice treated with the combined optimized treatment compared to IL-2:mAb complexes or tetramer treatment only, evidenced by both a polyclonal as well an antigen-specific stimulation (Supplementary Fig. 4A). Ag-specific T cell produced also IFN-γ. Remarkably, the combined optimized treatment produced significant less IFN-γ compared to the combined treatment (Supplemental Fig. 4B), which translated into an increased IL-10/IFN-γ ratio (Fig. 6B), indicating that either more IL-10 than IFN-γ secreting 2.5 mi^+^ T cells were present in the Ag-specific T cell population, or that IL-10 secretion dominated over IFN-γ secretion independently of the number of secreting T cells. In order to assess the *in vivo* suppressor activity, we treated NOD.Foxp3^EGFP^ mice with a combination of tetramers and IL-2:mAb complexes, with tetramers only, with IL-2:mAb complexes only, or left them untreated. Next, total splenocytes were transferred along with splenocytes from BDC-2.5/N TCR Tg mice containing diabetogenic T cells into NOD.SCID mice. Transfer of BDC-2.5 splenocytes led to 100% diabetes onset. This was not significantly different from 80% of diseased mice that had received splenocytes from either naive or tetramer-treated donors. To the contrary, less than 20% of recipients of splenocytes from mice treated either with IL-2:mAb complexes or IL-2:mAb complexes together with tetramers (combined optimized treatment) succumbed to T1D (Fig. 6C). Taken together, the combined optimized treatment as well as IL-2:mAb complexes led to solid protection against T1D, however, there was a quantitative difference in the IL-10 production between both treatments.

**Figure 7:**
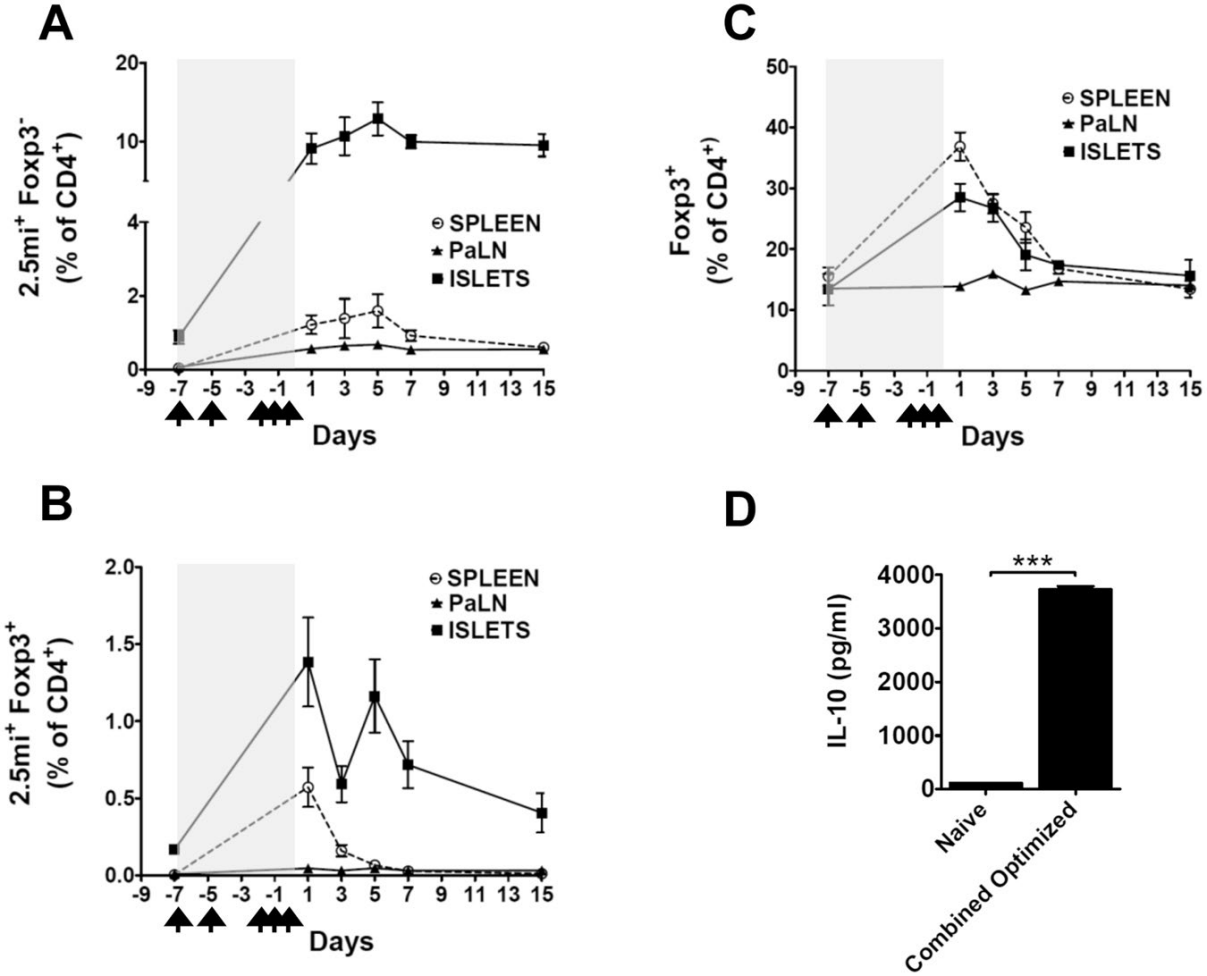
Time-course analysis of the polyclonal and antigen-specific Treg response to the combined optimized treatment. Unless otherwise indicated in the figure, analysis of the spleen is shown. Seven to 11 weeks-old NOD.Foxp3^EGFP^ animals (n = 3–6/time point) received the combined optimized treatment (arrows indicate days of immunization with A^g7^/2.5 mi tetramer and/or IL-2:mAb complexes and shaded areas inside the graphs indicated the whole duration of the treatment) and were sacrificed on day 1, 3, 5, 7 or 15 after the last injection. Spleen, pancreatic lymph nodes (PaLN) and pancreatic islets were isolated from mice and analyzed by tetramer staining. (**A**) Tconv (CD4^+^Foxp3^−^2.5 mi^+^) expansion. (**B**) Antigen-specific Treg (CD4^+^Foxp3^+^2.5 mi^+^) expansion. (**C**) Polyclonal Foxp3^+^CD4^+^ Treg expansion. Cells in (**A**,**B**) and (**C**) were gated on CD19^−^, CD8^−^, CD11c^−^, F4/80^−^, propidium iodide (PI)^−^ and CD4^+^. Mean ± SEM of two independent experiments is depicted. No A^g7^/GPI-specific T cell expansion was observed. (**D**) Sorted CD4^+^Foxp3^−^ T cells from 11 to 12 weeks-old treated (combined optimized treatment) or from naive NOD.Foxp3^EGFP^ mice were plated along with total splenocytes from 6–9 weeks-old NOD.SCID mice (as APC) and stimulated with 2.5 mi synthetic peptide. Supernatants were collected after 3 days and IL-10 quantified via ELISA. Data (mean ± SEM) is representative of two independent experiments; each point was collected in triplicate per experiment. ***p < 0.0005.

Next, we carried out a time-course analysis in NOD.Foxp3^EGFP^ mice that had received the combined optimized treatment. In the spleen and islets, Foxp3^−^2.5 mi^+^ T cells reached highest percentages at day 5. In the same tissues, percentages of Foxp3^+^2.5 mi^+^ T cells peaked at day 1 (Fig. 7A,B). At day 15, in the spleen Foxp3^−^2.5 mi^+^ T cells had declined to 0.5%, and 2.5 mi^+^Foxp3^+^ had retreated to baseline. To the contrary, around 10% of Foxp3^−^2.5 mi^+^ and 0.5% Foxp3^+^2.5 mi^+^ T cells remained in the islet at this time point, which was thus well above levels found in the spleen and the pancreatic LN. Total Foxp3^+^CD4 T cells peaked at day 1 but retracted to baseline levels by day 15 in all three tissues (Fig. 7C).

Given the augmented level of 2.5 mi^+^ Tconv, we wondered whether, at least partially, these cells could have regulatory function. We thus analyzed IL-10 production of isolated Foxp3^−^CD4^+^ T cells after stimulation with 2.5 mi peptide *in vitro* of NOD.Foxp3^EGFP^ mice that had received the optimized combined treatment. Antigen-specific T cells from treated mice produced significantly more IL-10 than from untreated controls (Fig. 7D). These cells therefore likely contribute to T1D suppression *in vivo*.

### Refining a long-term treatment protocol leads to solid protection against T1D

We next investigated how the combined optimized treatment protocol could be converted to a long-term treatment protocol in order to prevent T1D in NOD.Foxp3^EGFP^ mice. We first analyzed expression of CD25 in the 2.5 mi^+^Foxp3^−^ T cell population and found that its expression peaked at day 1 after the last immunization, but dropped to baseline at day 3 (not shown). Given the elevated percentages of Foxp3^+^ T cells at day 3 (Fig. 7B,C), we argued that this should be a good time point to continue with a second cycle of the same treatment. However, in mice receiving this second treatment at day 3, percentages of total 2.5 mi^+^ T cells as well as 2.5 mi^+^Foxp3^+^ T cells were reduced to one third or less in comparison to a single treatment cycle only (not shown). We suspected that this was due to T cell exhaustion resulting from a frequent tetramer injection in a short time span. We therefore established two treatment protocols, termed combined simple treatment and combined intensive treatment. In these treatment approaches, tetramers were injected every two weeks, in combination with more (combined intensive treatment) or less (combined simple treatment) IL-2:mAb complex injections (Supplementary Fig. 5A,B). 2.5 mi^+^ Tconv and 2.5 mi^+^Foxp3^+^ T cells were analyzed at weekly time points to measure the progression of both populations. Spacing of tetramer injection led to the expansion, contraction and re-expansion of 2.5 mi^+^ T cells in the spleen, therefore, the phenomenon of T cell exhaustion could be overcome with both approaches (Supplementary Fig. 6A). In comparison, the combined intensive treatment led to significantly more Foxp3^+^ T cells within the 2.5 mi^+^ T cell population than the combined simple treatment after week 2 (Supplementary Fig. 6B), at which time point more of these cells were CD25^hi^ (Supplementary Fig. 6C).

**Figure 8:**
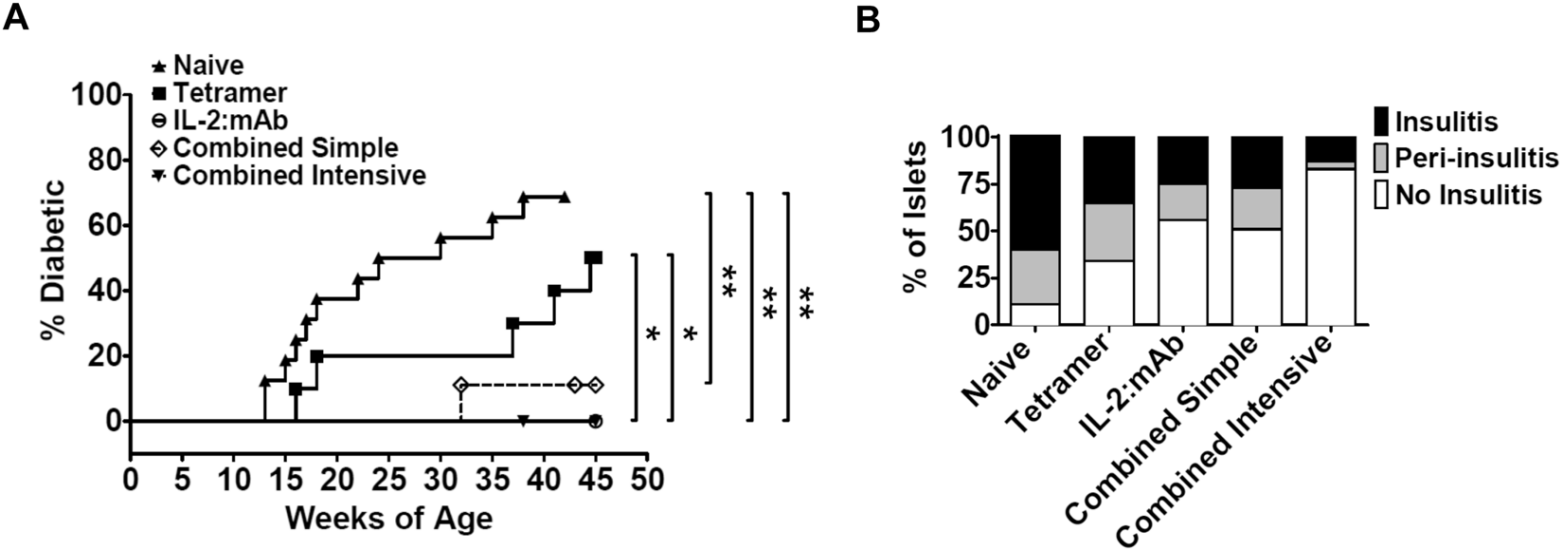
Cumulative incidence of diabetes and percentage of insulitis in NOD.Foxp3^EGFP^ females treated long-term with A^g7^/2.5 mi tetramer and/or IL-2:mAb complexes. (**A**) NOD.Foxp3^EGFP^ females received the indicated treatments starting at 5-6 weeks of age until week 35 (for further explanation of immunization protocol, see *Materials and Methods* and Supplemental Fig. 7). Tetramer group (n = 10), IL-2:mAb (n = 10), combined simple treatment (n = 9), combined intensive treatment (n = 10) and untreated (n = 16). Blood glucose was determined weekly; mice were considered diabetic after two consecutive measurements above 200 mg/dl. Mantel-Cox test was applied for statistical analysis (95% confidence). (**B**) Insulitis score of mice that remained free of T1D was analyzed by histology. The percentage of non-infiltrated, peri-infiltrated and totally infiltrated pancreatic islets is depicted for each treatment. Data for each group represent 100 islets from 3 mice.

Next, we carried out long-term T1D prevention studies in NOD.Foxp3^EGFP^ mice, that were, starting at 5-6 weeks-of-age, either left untreated or were treated until 35 weeks-of-age with tetramers only, IL-2:mAb complexes only, or one cycle of the combined optimized treatment followed by either the combined simple treatment or the combined intensive treatment (Supplementary Fig. 7). By week 37, 70% of untreated mice had become diabetic, and by week 45, 50% of tetramer only treated mice (Fig. 8A). To the contrary, only 10% of the mice receiving the combined simple treatment, and none of the mice receiving the combined intensive treatment or IL-2:mAb complexes only were diabetic. Therefore, the latter three treatments conferred solid protection for at least 10 weeks beyond the end of treatment. Importantly, the analysis of islet infiltration demonstrated that of all four treatment groups, insulitis was almost completely suppressed only by the intensive combined treatment, while close to 50% of either insulitis or peri-insulitis was detected in mice receiving either the combined simple or IL-2:mAb complex treatment only (Fig. 8B). Taken together, the optimization of the combinatorial tetramer/IL-2:mAb complexes treatment protocol, guided by tetramer analysis, led to the protection against insulitis and to a solid prevention of T1D.

## Discussion

We previously used different approaches, such as DNA treatment as well as the subunit B of cholera toxin (CTB), both designed to enhance presentation of the 2.5 mi peptide by APCs, in an effort to prevent disease in NOD mice. We found that the DNA treatment induced partial protection and did not observe the generation of antigen-specific Foxp3^+^ CD4 T cells^34^. To the contrary, the CTB-2.5 mi fusion protein given orally did result in the generation of antigen-specific Foxp3^+^ CD4 T cells, but treatment did not protect from T1D in our hands, and NOD mice failed to generate oral tolerance against the peptide^10^. We thus searched for alternative methods to induce highly functional antigen-specific Treg to mediate protection against this disease.

It has been previously shown that IL-2:mAb complex treatment can increase total Foxp3^+^ T cells^17^ and that treatment with MHC/peptide tetramers or MHC/peptide nanoparticles can induce Foxp3^−^ T cells with regulatory capacity^16,35^. Therefore, we anticipated that the combination of both approaches should have a beneficial synergistic effect. In NOD mice, low dose IL-2 treatment led to T1D prevention^12^, and a relative short treatment period with IL-2 or IL-2:mAb complexes has even been reported to reverse clinical diabetes^36^. However, the effect on antigen-specific T cells has not been analyzed in these studies. Our goal of the present work was to analyze quantitative and functional changes of these populations following a combination treatment consisting of MHC/peptide and IL-2:mAb complexes designed to maximize the *in vivo* generation of antigen-specific Foxp3 Treg.

We have shown that the 2.5 mi peptide, complexed to A^g7^ or in the DNA format, is able to generate tolerance in the polyclonal autoreactive T cell setting in the NOD mouse. In combination with A^g7^, several peptides from different autoantigens were individually able to do so, provided that they target a preexisting autoreactive T cell population since the use of a xenoantigen-derived peptide (hen egg lysozyme) did not blunt T1D^35^. This indicates that the choice of islet antigen used for therapy is likely secondary. However, regulatory T cells need to be generated that can reach the target organ and recognize autoantigen *in situ*^37^. Based on previous observations^13,38^, we predicted that the generation of autoantigen-specific Treg such as 2.5 mi^+^ Treg cells should have superior beneficial effects compared to the expansion of total Treg.

An advantage of the study of 2.5 mi^+^ T cells lies in the relatively large size of their population compared to other CD4 T cell populations that in some cases can only be quantified via a laborious enrichment procedure^39^. Here, the A^g7^/2.5 mi tetramer based analysis was key in developing step-by-step a treatment protocol of which we could optimize the timing of IL-2:mAb complex injection in combination with the tetramer treatment since this allowed detection of the time point when CD25 expression in the effector T cell population had been reduced to baseline while antigen-specific Treg were still expanding or at their maximum of expansion. These findings enabled us to fine tune treatment protocols, leading to an increment of the ratio of antigen-specific Treg to antigen-specific effector T cells from 1:15 in naive mice to 1:4 in immunized mice, and simultaneously increment the percentage of antigen-specific Foxp3^+^ Treg over 50-fold. Further, the tetramer analysis was essential to evaluate T cell exhaustion. We observed previously that a too frequent T cell stimulation *in vivo* with CTB-2.5 mi leads to a reversible exhaustion of this population (unpublished), and that the contraction of this population needs to be awaited before further stimulations can take place. Here, 2.5 mi^+^Foxp3^+^ Treg were reduced after two weeks to baseline values in spleen and PaLN but not in the islets. To the contrary, the antigen-specific Foxp3^−^ T cells population contracted only partially. A very high percentage was reached especially in the islets that persisted for at least two weeks, the maximal time span of our analysis. This is possibly due to the observed expression of CXCR3 by these cells that would facilitate their recruitment to the islets^32,33^, and additionally due to the presence of presented antigen at this site.

If diabetes prevention was the only criteria to evaluate the combined treatment versus treatment with IL-2:mAb complexes only, our approach seemingly did not lead to an improvement. However, both treatments were not equivalent as we observed two important quantitative differences. First, the combination of the tetramer together with IL-2:mAb complexes led to the production of IL-10 by antigen-specific T cells that clearly exceeded individual treatment with either reagent. We detected IL-10 production also in the 2.5 mi^+^Foxp3^−^ CD4 T cell population, something that has been observed previously by others^16,40,41^. Therefore, the expansion of both, Foxp3^+^ and Foxp3^−^ T cells, are likely beneficial in our approach. The ratio between IL-10 and IFN-γ secretion is superior in the combined and combined optimized treatment in comparison to IL-2:mAb complexes only which would suggest that the anti-inflammatory cytokine might keep effector T cells better in check. Nevertheless, IFN-γ might even be needed for Tregs to prevent T1D^42^. Second, the islet infiltration was close to absent in mice that had received the combined intensive treatment while all other treatments led to islet infiltrations close to or succeeding 50%, including the treatment with IL-2:mAb complexes only. Based on these findings, it is reasonable to predict that T1D prevention with the combined intensive treatment likely has a longer lasting protective effect than IL-2:mAb complexes only.

Moreover, the combined intensive treatment protected mice 10 weeks beyond termination of the long-term treatment. The relatively long period of protection without treatment indicates that it might be interrupted periodically for a prolonged period of time which opens a window to simplify this treatment in the future. We did not detect elevated percentages of 2.5 mi^+^Foxp3^+^ T cells in the spleen of these mice at this time point, while significantly more 2.5 mi^+^Foxp3^−^ T cells were found in mice receiving tetramer, the combined simple or the combined intensive treatment in comparison to treatment with IL-2:mAb complexes only (not shown). This is to be expected, however, since the maintenance of Ag-specific Foxp3^+^ T cell needs periodical Ag contact and elevated amounts of IL-2. Even though, it is possible that both cell types are maintained for prolong time periods in islets as this is where their Ag is present. An indication that this may take place is our time course analysis (Fig. 7): two weeks after treatment, the highest Ag-specific T cell percentages are found in the islets.

We here show that the combination of A^g7^/peptide tetramers with IL-2:mAb complexes has a robust capability to prevent T1D, even in the accelerated disease model comprising the transfer of BDC-2.5 splenocytes into NOD.SCID mice. A different report has recently shown that IL-2:mAb complexes combined with a BDC-2.5 activating mimotope, different from the one used by us here, and rapamycin prevent T1D^43^. The authors detected IL-4 as well as IL-10 production, leading to the conclusion that a TH2 profile had been generated apart from islet antigen-specific CD4 T cells and the expansion of total Treg. Islet infiltration was 50% in the study and thus higher than in our approach. A combination of rapamycin with low dose IL-2 has also been reported by a different group but the protection did not reach 100%^44^. However, counterproductive effects on the use of a combined IL-2/rapamycin therapy in the NOD mouse have been published^45^. In T1D patients, this combination therapy had positive effects on the number of Treg but clinical data in all subjects worsened during the trial^46^. Further trials thus are necessary to establish whether IL-2 together with rapamycin alone has a chance to lead to clinical benefits. Two encouraging clinical studies on the use of low dose IL-2 in T1D patients have shown that low-dose IL-2 is reasonably well tolerated and that the number of Treg increase in these patients^47,48^. This was accompanied with the upregulation of activation markers in the Treg population and an increase in IL-10 and TGF-β production. The author also detected an increase of IL-17 and IFN-γ production at some doses, the latter a parallel to our own findings. This underlines the complexity of the immune response following IL-2 therapy. The production of pro-inflammatory cytokines is not necessarily contraproductive as long as the anti-inflammatory response is dominant.

In conclusion, our study is an important step towards a solid long-term protection against T1D. The combined treatment expands polyclonal as well as antigen-specific Foxp3^+^ regulatory T cells, with a limited life span. However, it also leads to the expansion of antigen-specific Foxp3^−^ but IL-10 producing T cells that persist over prolonged periods of time, especially in the islet. Both responses are likely to work in concert, leading to complete diabetes prevention and minimal islet infiltration. How Ag-specific Foxp3- Treg are generated by our treatment is still unclear. Long-term tetramer treatment alone is less efficient in regards to T1D prevention but it has a positive effect. Tetramer alone therefore might expand preexisting IL-10 producing Foxp3^−^ Treg. In addition, the presence of IL-2:mAb complexes likely generates a regulatory milieu in which the increased presence of polyclonal and Ag-specific Foxp3^+^ Treg might lead to the generation of tolerogenic dendritic cells. In turn, these dendritic cells may generate de novo Ag-specific Foxp3^−^ Treg producing additional IL-10 from naive T cells. These T cells, after their priming by dendritic cells, could come in contact with soluble tetramer, which may lead to their further proliferation. The next step is to test this approach in recent-onset diabetic mice. Augmented numbers of islet-antigen specific T cells generated during the disease process might help to increase the efficacy of the treatment. Our approach should be helpful to pave the road for equivalent treatment strategies in human subjects, especially when taking into account the presence of autoreactive T cells against chromogranin A in T1D patients. It also underlines the necessity to use MHC tetramers in clinical studies in order to collect the corresponding data as they will allow drawing educated conclusions that can speed up the race towards developing sturdy treatment protocols for the clinic.

## Electronic supplementary material

Supplemental Information
