# Supplementary material for: Treatment of T1D via optimized expansion of antigen-specific Tregs induced by IL-2/anti-IL-2 monoclonal antibody complexes and peptide/MHC tetramers
Source: Sci Rep. 2018 May 25;8:8106. doi: 10.1038/s41598-018-26161-6 (PMC5970271; doi:10.1038/s41598-018-26161-6)
Supplement: Supplementary file 1 — Supplemental Information [file 41598_2018_26161_MOESM1_ESM.pdf]

Treatment of T1D via optimized expansion of antigen-specific Tregs induced by IL-2/anti-IL-2 monoclonal antibody complexes and peptide/MHC tetramers

Cristina Izquierdo[*], Angela Zarama Ortiz[*,†], Maximiliano Presa[*,‡], Sara Malo[*], Anna Montoya[*,§], Nahir Garabatos[*,¶], Conchi Mora[||], Joan Verdaguer[||,#], and Thomas Stratmann[*,**]

[*]Department of Cell Biology, Physiology and Immunology, Faculty of Biology, University of Barcelona, 08028 Barcelona, Spain
[†]Current Affiliation: Otsuka Pharmaceutical, S.A., Barcelona, Spain
[‡]Current Affiliation: The Jackson Laboratory, Bar Harbor, USA
[§]Current Affiliation: Danone Nutricia, Madrid, Spain
[¶]Current Affiliation: Benaroya Research Institute, Seattle, USA
[||]Immunology Unit, Department of Experimental Medicine, School of Medicine, University of Lleida and IRB Lleida, 25008 Lleida, Spain
[#]CIBER of Diabetes and Associated Metabolic Diseases (CIBERDEM)

[**] Corresponding author:
Department of Cell Biology, Physiology and Immunology, Faculty of Biology, University of Barcelona, Avenida Diagonal 643, 08028 Barcelona, Spain
Phone number +34 934021533, Fax number +34 934110358, e-mail: thomas.stratmann@ub.edu

Supplementary Figures.

Supplemental Table 1. Fold changes in the percentage of Ag-specific T cell populations found in spleen and islets of NOD.Foxp3^{EGFP} mice after the administration of the indicated treatments.

		Naive*	IL-2:mAb*	Tetramer*	Combined*	Combined Optimized*
**Percentage/fold increase in 2.5mi^{+} Foxp3^{+} cells**	**SPLEEN**	0,003% / x1	0,007% / x2,18	0,047% / x14,89	0,243% / x76,90	0,172% / x54,27
	**ISLETS**	0,190% / x1	0,245% / x1,29	0,374% / x1,97	0,828% / x4,36	1,378% / x7,25
**Percentage/fold increase in 2.5mi^{+} Foxp3^{-} cells**	**SPLEEN**	0,036% / x1	0,032% / x0,87	0,821% / x22,60	1,992% / x54,83	0,720% / x19,82
	**ISLETS**	1,321% / x1	0,877% / x0,66	3,966% / x3,00	7,598% / x5,75	7,769% / x5,88

*Data shown in the table originate from experiments described in Figures 1-4. Mean percentages were calculated for each treatment and type of cell including data obtained in all the experiments done (n=9-12 per treatment). Values depicted in the table were obtained dividing the mean percentage of cells in the indicated treatments by the mean percentage of the same population found in naive mice (note that for calculations the exact numbers were used while in the table rounded numbers are presented). Percentages refer to 2.5mi^{+}Foxp3^{+} or 2.5mi^{+}Foxp3^{-} within total CD4^{+} T cells.

Supplemental Table 2. Fold changes in the percentage of CD25^{hi} Ag-specific T cell populations found in spleen and islets of NOD.Foxp3^{EGFP} mice after the administration of the indicated treatments.

		**Naive***	**IL-2:mAb***	**Tetramer***	**Combined***	**Combined Optimized***
**Percentage/fold increase in 2.5mi$^+$ Foxp3$^+$ CD25^{hi} cells**	**SPLEEN**	0,0008% x1	0,0043% x5,10	0,0165% x19,66	0,2333% x277,37	0,1603% x190,58
	**ISLETS**	0,0266% x1	0,1703% x6,41	0,1201% x4,52	0,7623% x28,70	1,2160% x45,78
**Percentage/fold increase in 2.5mi$^+$ Foxp3$^-$ CD25^{hi} cells**	**SPLEEN**	0,0001% x1	0,0001% x1,01	0,0075% x67,00	1,1080% x9910,55	0,1440% x1288,01
	**ISLETS**	0,0317% x1	0,0114% x0,36	0,0832% x2,62	2,8170% x88,78	1,2930% x40,75

*Data shown in the table originate from experiments described in Figures 1-4. Mean percentages were calculated for each treatment and type of cell including data obtained in all the experiments done (n=9-12 per treatment). Values depicted in the table were obtained dividing the mean percentage of cells in the indicated treatments by the mean percentage of the same population found in naive mice (note that for calculations the exact numbers were used while in the table rounded numbers are presented). Percentages refer to 2.5mi$^+$Foxp3$^+$CD25^{hi} or 2.5mi$^+$Foxp3$^-$CD25^{hi} within total CD4$^+$ T cells.

Supplemental Table 3. Expansion of Treg Foxp3^{+} populations in pancreatic islets induced by the combined optimized treatment.

		Naive*	Combined Optimized*	Increase Ratio
**Treg cells (% of CD4^{+})**	**Polyclonal**	21.5% ± 1.3	31.1% ± 1.6	x 1.45
	**2.5mi^{+}**	0.19% ± 0.04	1.38% ± 0.26	x 7.25

* Values are mean ± SEM. Each group include 9-12 animals.

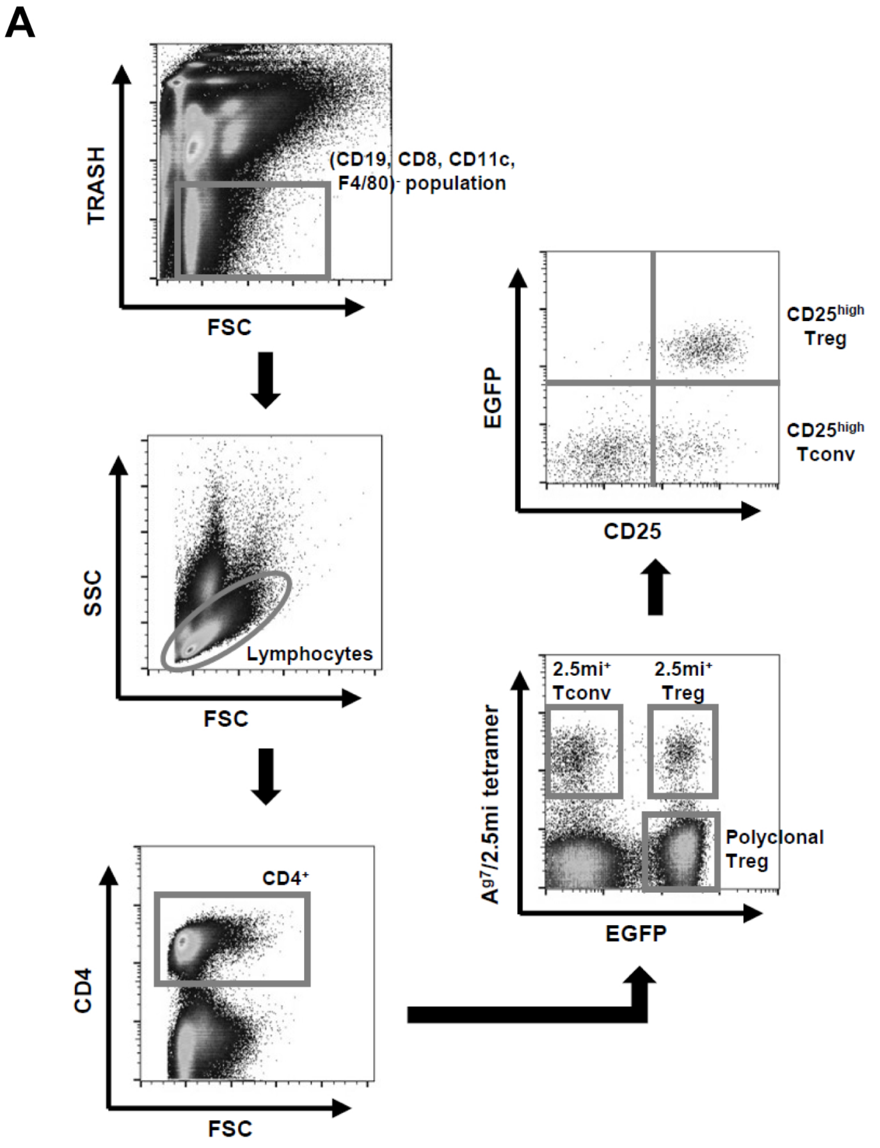

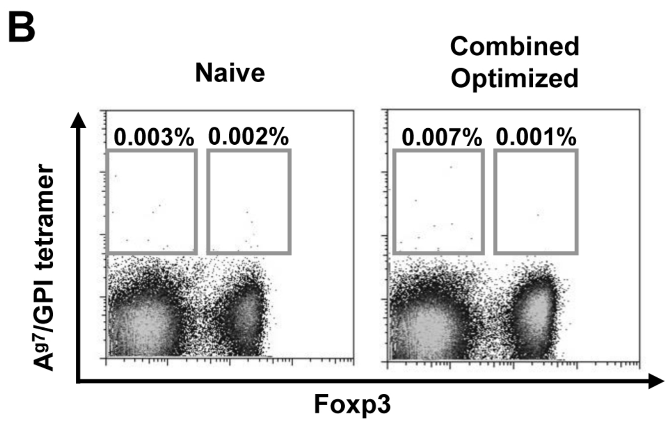

Supplementary Figure 1. Gating strategy applied in flow cytometry files for the analysis of 2.5mi^{+} T cells in NOD.Foxp3^{EGFP} reporter mice. (**A**) CD19^{+}, CD11c^{+}, CD8^{+}, F4/80^{+} (stained with PECy5 antibodies) and dead cell populations (containing propidium iodide) were excluded during the analysis. Lymphocytes were next select via SSC/FSC analysis, followed by gating on CD4^{+} T cells. Treg and Tconv 2.5mi^{+} T cells within CD4^{+} T cells were detected via tetramers versus EGFP (= Foxp3) analysis. Depending on the analysis, Ag-specific as well as total CD4^{+}Foxp3^{+} T cells were further analyzed for their expression of CD25. The sample used in the example originates from a spleen of a mouse receiving the combined optimized treatment. (**B**) Representative example of an A^{g7}/GPI tetramer control staining of splenocytes derived from a naive mouse (left) and a mouse receiving the combined optimized treatment (right). Cells were gated on total CD4^{+}.

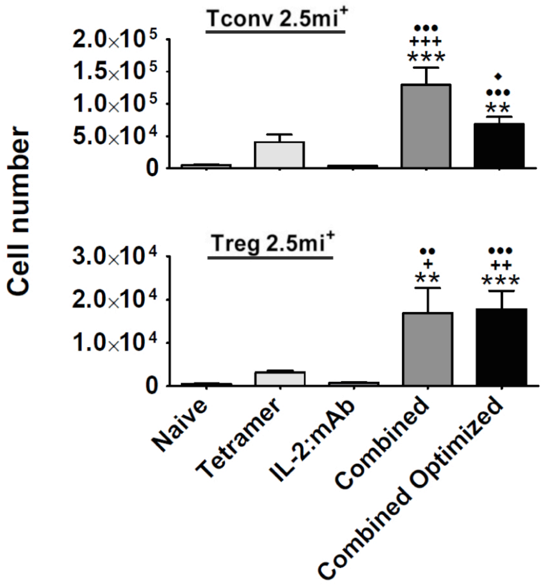

Supplementary Figure 2. Antigen-specific T CD4+ cells counts in spleen after the administration of protein treatments. Three to six NOD.Foxp3EGFP mice received the indicated treatments i.p. following the same immunization protocols described in figures 1 and 3. Spleens were harvested and homogenized, splenocytes were counted using a Neubauer chamber and also analyzed by tetramer staining by flow cytometry. Number of cells were calculated using total cell numbers counted and the percentage of 2.5mi+ cells (Tconv and Treg) obtained in the cytometric profile of each organ. Mean ± SEM is shown in graphs. *, vs. naive; +, vs. only tetramer treatment; ●, vs. IL-2:mAb complexes treatment; ♦, vs. combined treatment. $^{**}p < 0.005$, $^{***}p < 0.0005$.

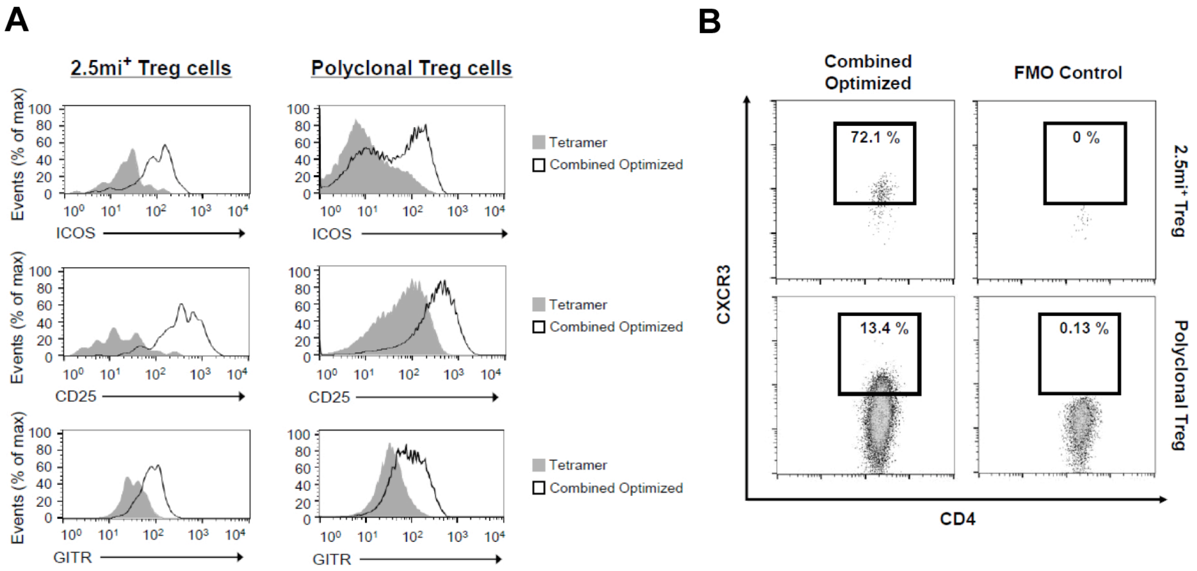

Supplementary Figure 3. Phenotype surface markers on regulatory T cells expanded with the combined optimized treatment. Analysis of the spleen are shown. (**A**) Representative histograms of the expression of ICOS, CD25 and GITR on the surface of 2.5mi$^+$ (left) and polyclonal (right) Foxp3$^+$CD4$^+$ T cells in NOD.Foxp3^{EGFP} females that received either the combined optimized treatment or A^{g7}/2.5mi tetramer injections only. (**B**) Representative dot plot showing the percentages of CXCR3$^+$ cells within antigen-specific (top) or polyclonal (bottom) Foxp3$^+$CD4$^+$ T populations of the same treated animals analyzed in A. 2.5 mi$^+$ cells were excluded from polyclonal T cells for all the analysis. Data shown in this figure are representative examples from 3 independent experiments; n = 2-3 animals/experimental group.

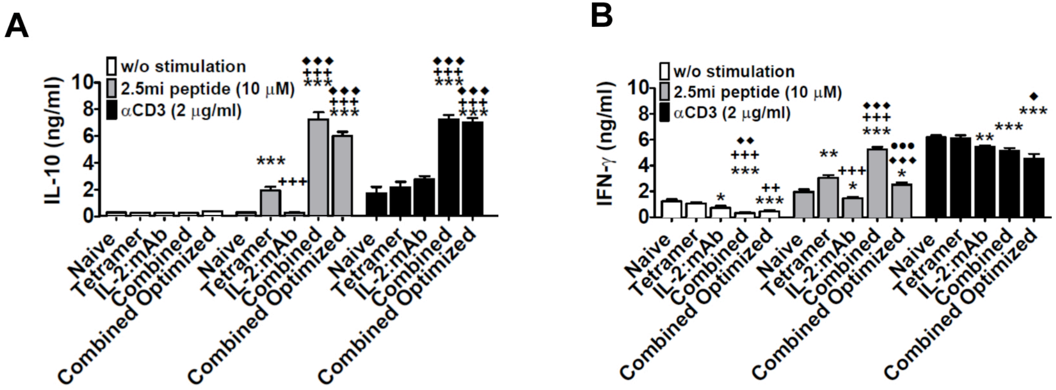

Supplementary Figure 4. IL-10 and IFN-γ production in splenocyte cultures from NOD.Foxp3[EGFP] females treated with combined protein treatments. Data used for the calculation of IL-10/IFN-γ ratio in figure 6 is depicted here. (**A**) IL-10 secretion detected in spleen cultures after 3 days of stimulation. (**B**) IFN-γ presence in the same cellular cultures. Statistical differences were calculated using Student t test applying a 95% confidence interval. *, vs. naive; +, vs. only tetramer treatment; ♦, vs. IL-2:mAb complexes treatment; ●, vs. combined treatment.

A COMBINED SIMPLE

1 week

1	2	3	4	5	6	7
2.5mi tet			IL-2:mAb	**ANALYSIS**		

2 weeks

1	2	3	4	5	6	7
2.5mi tet			IL-2:mAb			
-			IL-2:mAb	**ANALYSIS**		

3 weeks

1	2	3	4	5	6	7
2.5mi tet			IL-2:mAb			
-			IL-2:mAb			
2.5mi tet			IL-2:mAb	**ANALYSIS**		

B COMBINED INTENSIVE

1 week

1	2	3	4	5	6	7
2.5mi tet			IL-2:mAb	**ANALYSIS**		

2 weeks

1	2	3	4	5	6	7
2.5mi tet			IL-2:mAb			
IL-2:mAb				IL-2:mAb	**ANALYSIS**	

3 weeks

1	2	3	4	5	6	7
2.5mi tet			IL-2:mAb			
IL-2:mAb				IL-2:mAb		
2.5mi tet			IL-2:mAb	**ANALYSIS**		

Supplementary Figure 5. Immunization schedule to test the efficiency of the combined simple and the combined intensive maintenance treatments designed for long-term T1D prevention studies. The weekly injection protocols for the Combined Simple (**A;** green) and the Combined Intensive (**B;** red) treatments are shown. Differences between treatments have been highlighted in color. NOD.Foxp3^{EGFP} mice were treated for either one, two or three weeks. *2.5mi tet*: 25 µg of 2.5mi MHC tetramer were administered i.p.; *IL-2:mAb*: 1 µg of interleukin 2 bound to 5 µg of JES6-1A12 mAb were injected i.p..

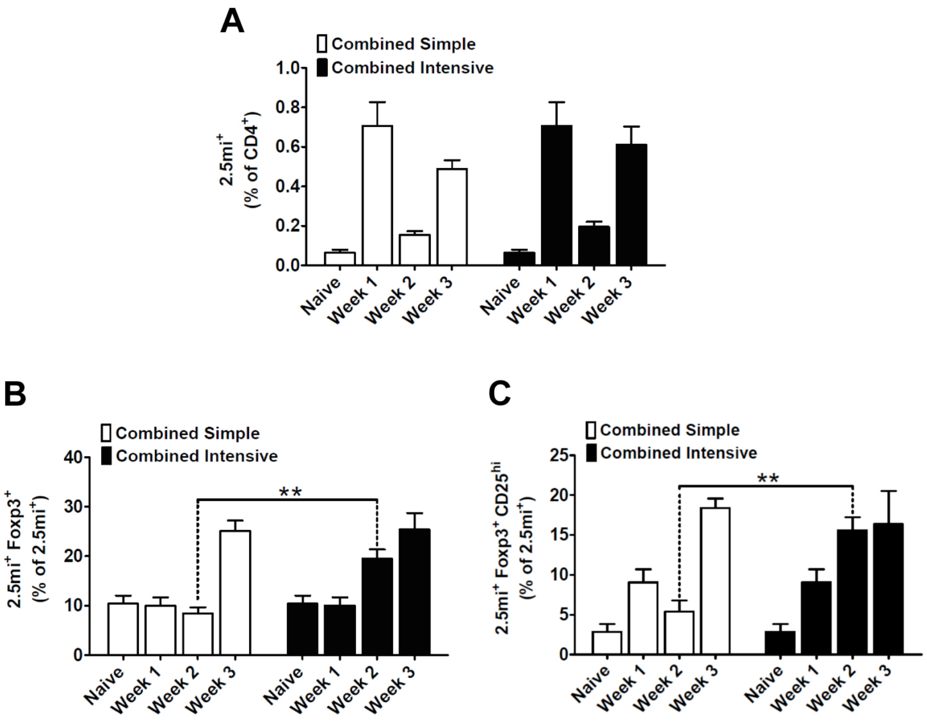

Supplementary Figure 6. Antigen-specific T cell response in NOD.Foxp3^{EGFP} mice after receiving the combined simple or the combined intensive treatment during a three-week period. 5 to 7 NOD.Foxp3^{EGFP} mice (8 to 13 weeks-old) per experimental group were treated as explained in Supplemental Figure 5 or left untreated. At the indicated end-point mice were sacrificed and cellular suspensions of their spleen were analyzed by flow cytometry using tetramers. (**A**) Antigen-specific T cell (2.5mi^{+}CD4^{+}) response for each experimental group and treatment (percentages are calculated within total CD4^{+} T cells). (**B**) 2.5mi^{+} Treg cells (T CD4^{+}2.5mi^{+}Foxp3^{+}) analyzed within total 2.5mi^{+} T cell population. (**C**) CD25 expression on 2.5mi^{+} Treg cells. Mean ± SEM of data obtained in two independent experiments is depicted. $^{**}p < 0.005$, determined by Student t test.

COMBINED INTENSIVE TREATMENT

Day	1	2	3	4	5	6	7
**WEEK 1**	2.5mi tet	-	2.5mi tet	-	-	IL-2:mAb	IL-2:mAb
**WEEK 2**	IL-2:mAb	-	-	-	IL-2:mAb	-	-
**WEEK 3**	2.5mi tet	-	-	IL-2:mAb	-	-	-
**WEEK 4**	IL-2:mAb	-	-	-	IL-2:mAb	-	-

MAINTENANCE DOSING (weeks 3–4) **(Administered until week 35 of age)**

COMBINED SIMPLE TREATMENT

Day	1	2	3	4	5	6	7
**WEEK 1**	2.5mi tet	-	2.5mi tet	-	-	IL-2:mAb	IL-2:mAb
**WEEK 2**	IL-2:mAb	-	-	-	IL-2:mAb	-	-
**WEEK 3**	2.5mi tet	-	-	IL-2:mAb	-	-	-
**WEEK 4**	-	-	-	IL-2:mAb	-	-	-

MAINTENANCE DOSING (weeks 3–4) **(Administered until week 35 of age)**

IL-2:mAb TREATMENT

Day	1	2	3	4	5	6	7
**WEEK 1**	-	-	-	-	-	IL-2:mAb	IL-2:mAb
**WEEK 2**	IL-2:mAb	-	-	-	IL-2:mAb	-	-
**WEEK 3**	-	-	-	IL-2:mAb	-	-	-
**WEEK 4**	IL-2:mAb	-	-	-	IL-2:mAb	-	-

MAINTENANCE DOSING (weeks 3–4) **(Administered until week 35 of age)**

pMHC TETRAMER TREATMENT

Day	1	2	3	4	5	6	7
**WEEK 1**	2.5mi tet	-	2.5mi tet	-	-	-	-
**WEEK 2**	-	-	-	-	-	-	-
**WEEK 3**	2.5mi tet	-	-	-	-	-	-
**WEEK 4**	-	-	-	-	-	-	-

MAINTENANCE DOSING (weeks 3–4) **(Administered until week 35 of age)**

Supplementary Figure 7. Immunization schedules for long-term T1D preventions studies. The immunization schedule for each experimental group assayed is shown in the figure: NOD.Foxp3^{EGFP} females were treated starting at 5-6 weeks-of-age; during the first two weeks mice received the combined optimized treatment (first two tables) or just IL-2:mAb complexes or A^{g7}/2.5mi tetramers following the same schedule as in combined treatment (last two tables). After the first boost of cells mice received a maintenance dosing (weeks 3 and 4 in tables) to keep regulatory T cells in high numbers. The maintenance schedule depicted was repeated until week 35 of age of animals. Final analysis was carried out at 45 weeks-of-age. *2.5mi tet*: 25 μg of mimotope tetramer were administered i.p.; *IL-2:mAb*: 1 μg of interleukin 2 bound to 5 μg of JES6-1A12 mAb were injected i.p..
